# Pain Management and Rehabilitation for Central Sensitization in Temporomandibular Disorders: A Comprehensive Review

**DOI:** 10.3390/ijms232012164

**Published:** 2022-10-12

**Authors:** Martina Ferrillo, Amerigo Giudice, Nicola Marotta, Francesco Fortunato, Daniela Di Venere, Antonio Ammendolia, Pietro Fiore, Alessandro de Sire

**Affiliations:** 1 Dentistry Unit, Department of Health Sciences, University of Catanzaro “Magna Graecia”, 88100 Catanzaro, Italy; 2 Physical Medicine and Rehabilitation Unit, Department of Medical and Surgical Sciences, University of Catanzaro “Magna Graecia”, 88100 Catanzaro, Italy; 3 Institute of Neurology, Department of Medical and Surgical Sciences, University of Catanzaro “Magna Graecia”, 88100 Catanzaro, Italy; 4 Department of Interdisciplinary Medicine, University of Bari “Aldo Moro”, 70124 Bari, Italy; 5 Neurological Rehabilitation Unit, Istituti Clinici Scientifici Maugeri, IRCCS Institute of Bari, 70124 Bari, Italy; 6 Department of Clinical and Experimental Medicine, University of Foggia, 71100 Foggia, Italy

**Keywords:** temporomandibular disorders, central sensitization, pain, myofascial pain, rehabilitation

## Abstract

Temporomandibular disorders (TMD) are a group of musculoskeletal diseases affecting masticatory muscles and temporomandibular joints (TMJ). In this context, the chronic TMD could be considered as a condition with chronic primary orofacial pain, presenting as myofascial TMD pain or TMJ arthralgia. In this context, myogenous TMD may present overlapping features with other disorders, such as fibromyalgia and primary headaches, characterized by chronic primary pain related to dysfunction of the central nervous system (CNS), probably through the central sensitization. This phenomenon could be defined as an amplified response of the CNS to sensory stimuli and peripheral nociceptive, characterized by hyperexcitability in the dorsal horn neurons in the spinal cord, which ascend through the spinothalamic tract. The main objectives of the management of TMD patients are: decreasing pain, increasing TMJ function, and reducing the reflex masticatory muscle spasm/pain. The first-line treatments are physical therapy, pharmacological drugs, occlusal splints, laser therapy, extracorporeal shockwave therapy, transcutaneous electrical nerve stimulation, and oxygen–ozone therapy. Although all these therapeutic approaches were shown to have a positive impact on the central sensitization of TMD pain, there is still no agreement on this topic in the scientific literature. Thus, in this comprehensive review, we aimed at evaluating the evidence on pain management and rehabilitation for the central sensitization in TMD patients.

## 1. Introduction

Temporomandibular disorders (TMD) are a group of musculoskeletal and neuromuscular conditions affecting the masticatory muscles, the temporomandibular joint (TMJ), and the other associated structures [1]. According to the Diagnostic Criteria for TMD (DC/TMD) Axis I, TMD could be divided in intra-articular disorders, including disc displacement, arthralgia, arthritis, and arthrosis, and muscle disorders [1]. These latter are also defined as “myogenous TMD”, which can be further categorized into: local myalgia, if the pain is localized during palpation; myofascial pain, if the pain spreads within the palpated muscular territory; and myofascial pain with referral, if the pain spreads beyond the boundary of the masticatory muscles [1]. A recent systematic review and meta-analysis, with a combined sample of 2518 subjects, suggested that the prevalence of TMD could range from 25.2% to 34.9%, with a predominance of myofascial pain diagnosis (10.3–15.4%) [2]. The etiology is not clear, and it has been accepted as multifactorial, considering the multitude of initiating, predisposing, or perpetuating risk factors, including postural and parafunctional habits, repetitive microtrauma, direct and indirect trauma, and psychological factors, such as depression and anxiety [3]. The persistent and recurrent pain generated by the myofascial pain may cause limitations in the main activities of daily living (ADLs) and reduce the oral health-related quality of life (OHRQoL) [4]. If odontogenic causes are excluded [5,6], the painful TMD could be considered as the main cause of pain in the orofacial region [2,4].

More in detail, according to the 11th version of the International Classification of Diseases (ICD-11), the chronic primary pain was described as “pain in one or more anatomical regions that persists or recurs for longer than 3 months; is associated with significant emotional distress and/or significant functional disability; and the symptoms are not better accounted by another diagnosis” [7,8]. In this context, chronic TMD could be considered as conditions with chronic primary orofacial pain, presenting as myofascial TMD pain or TMJ arthralgia [7,8]. Compared to arthrogenous TMD, which appears to be a localized phenomenon, myogenous TMD may present overlapping features with other disorders, such as fibromyalgia and primary headaches, characterized by chronic primary pain related to dysfunction of the central nervous system (CNS), probably through the phenomenon of central sensitization [9,10,11,12,13].

Central sensitization can be defined as an amplified response of the CNS to sensory stimuli and peripheral nociceptive, characterized by hyperexcitability in the dorsal horn neurons in the spinal cord, which ascend through the spinothalamic tract [14,15,16,17]. Central sensitization could lead to the development of increased pain sensation from noxious stimuli, known as hypersensitivity, or pain originating from non-noxious stimuli, known as allodynia [14]. Moreover, other clinical features of central sensitization could be: increased temporal pain, summation spontaneous pain, referred pain, and pressure hyperalgesia [14,18].

Thus, the central sensitization could represent the basis of chronic pain “or pain that persists beyond a normal time of healing” in patients affected by TMD [19,20]. In the scientific literature, the central sensitization showed to have a role not only in the pathophysiology of TMD but also in other several chronic pain conditions, including: fibromyalgia, migraine, tension-type headache, irritable bowel syndrome, and chronic fatigue syndrome [21,22,23].

The psychological component in terms of emotional distress should be considered during the diagnosis process to better manage chronic pain conditions [8]. Anxiety, frustration, and depression may contribute to the development and to the persistence and exacerbation of pain [7,8].

Concerning the management of patients with TMD, it should be taken into consideration that the main objectives are: decreasing pain, increasing TMJ function, and reducing reflex masticatory muscle spasm/pain [24]. The first-line treatment for TMD is considered the conservative approach [25,26], including physical therapy [27], biofeedback [28], pharmacological drugs [29], TMJ injections [30], occlusal splints [31,32], laser therapy [33], extracorporeal shockwave therapy (ESWT) [34], transcutaneous electrical nerve stimulation (TENS) [35], and oxygen–ozone therapy [36].

**Figure 1:**
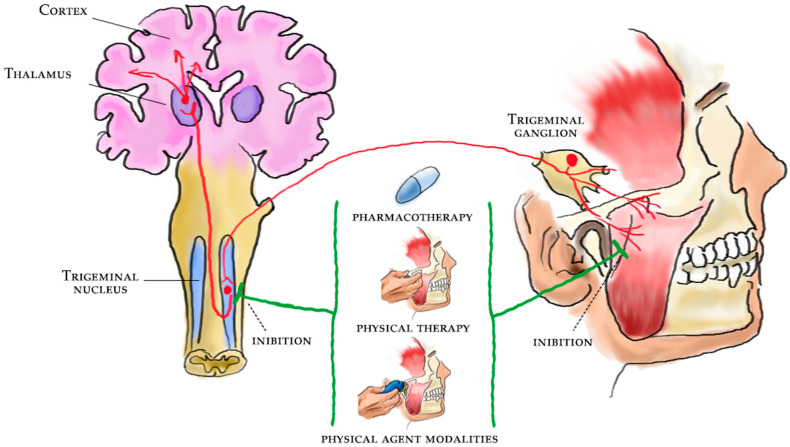
Pain management and rehabilitation for the central sensitization of temporomandibular disorders.

All these therapeutic approaches might have a positive impact on the central sensitization of TMD pain (see Figure 1), albeit there is still no agreement in the scientific literature on this topic in terms of the management of these peculiar patients.

It should be noted that myogenous TMD might present a chronic primary pain related to a CNS dysfunction due to the mechanism of central sensitization that could lead to hypersensitivity in TMD patients [14]. To date, the scientific literature still lacks strong evidence on the key role of central sensitization for chronic pain in TMD patients affected by TMD [19,20], probably because this phenomenon showed to have a role in the pathophysiology of other chronic diseases [21,22,23]. Therefore, the diagnosis and the treatment of the central sensitization should be better investigated, taking into account the positive results that some conservative approaches might have on the central sensitization of TMD pain [25,26,27,28,29,30,31,32,33,34,35,36].

In this context, by the present comprehensive review, we aimed to investigate the state-of-the-art therapies regarding pain management and rehabilitation for the central sensitization in TMD patients in order to adequately manage this detrimental condition.

## 2. Central Sensitization

In a neurophysiological scenario, two main physiological phenomena, namely central sensitization and impairment of the inhibitory system of descending pain, together with the physiological mechanism of neuronal convergence, might be considered fundamental factors explaining TMD clinical patterns of and the associations with other comorbidities [15,24]. Other important mechanisms, such as central facilitation, peripheral sensitization, and neuroimmune alterations, also contribute to the physiological frame and rationale underlying the coexistence of TMD and painful conditions [15,24]. Furthermore, the nociceptive pathways responsible for TMD and headache are similar; in fact, there is an important area in the association between these disorders: the spinal trigeminalis nucleus—in particular, the caudal subnucleus [37]. This region is primarily responsible for nociceptive input from the head and face; therefore, it could be considered the first “meeting point” between TMD and headache disorders [38]. The generic term central sensitization is all neuronal alterations that may follow nociceptive processing within the caudal subnucleus in patients with chronic trigeminal pain. In short, this phenomenon may refer to a group of changes in the arrangement and quantity of membrane channels and neurotransmitters that ultimately decrease neuronal threshold activation, increase the firing rate, and widen receptor fields [39].

Many mechanisms are involved in central sensitization, but two main events (i.e., activation of the N-methyl-D-aspartate receptor and inhibition of gamma aminobutyric acid (GABA) and glycine receptors) are assumed to be present in most chronic pain conditions [40,41]. Furthermore, additional neurotransmitters, e.g., substance P (SP) and the calcitonin gene-related peptide, both released by small fiber neurons, relate to the lasting depolarization of the neuronal membrane and the sum of nociceptive inputs [42]. In addition to this hyperexcitable plasticity of the central nervous system, some mechanisms include basic inhibitory activity and, consequently, facilitate nociceptive signaling. Inhibition of the activity of GABAergic and glycinergic interneurons is assumed to reduce this second inhibitory activity, which, in turn, can increase the depolarization and excitation of order neurons [43]. Positive feedback circuits can amplify these large, second-order central changes: dynamic range neurons. Finally, this state of hyperexcitability could be a gradual and frequency-dependent facilitation of the nociceptive (wind-up phenomena) states of chronic pain [44].

## 3. Diagnosis of Central Sensitization in TMD

The diagnosis of the central sensitization is mandatory for adequate management, and, at the same time, it is considered a challenge that should be overcome in the near future. The central sensitization is an important aspect that is involved in the pathophysiology of different musculoskeletal painful conditions, including TMD [45]. Although the central sensitization could not be directly measured, different assessment tools have been developed to measure the sensory experiences that are greater than expected in amplitude, duration, or spatial extent [46].

### 3.1. Algometry and Pressure Pain Thresholds

In this scenario, a pressure algometer could be an effective tool in the screening and evaluation of patients with muscle pain due to central sensitization [47]. Pressure algometry is predominantly a manual procedure that requires a perceptual response from the participant or patient and is commonly used in quantitative sensory testing (QST), used extensively in clinical and experimental pain studies [48]. More in detail, the pressure (i.e., force per area often expressed in kPa) that the participants first perceive to be painful is defined as the pain threshold, and the maximum pressure endured by the participants is defined as the tolerance threshold.

In this context, the pressure pain threshold (PPT), defined as the minimum amount of pressure capable of inducing pain, is also frequently used in the evaluation of hyperalgesia [49]. The reliability of PPT depends not only on the application technique of the observer but also on the ability of the patient or participant to provide a consistent verbal indication of the PPT level [50]. The simplest algometry type allows the assessment of pressure using pressure-sensitive devices fitted to the finger [51]. Hand-held devices based on spring coil systems are also frequently used [52]. More sophisticated pressure algometers commonly provide visual feedback on the pressure application rate, which has been shown to influence the pain threshold [53]. In this scenario, it was demonstrated that patients with dysfunctions of the masticatory system showed variations in the repeatability of the pain threshold during pressure algometry [54]. More specifically, in TMD patients, the algometer can be applied to the masseter and temporal muscles and to the lateral pole of the condyle, recording the measurement in Kg when the patient felt pain or placing the algometer to measure 1 cm^2^ in direct contact with the joint and applied increasing pressure until the patient reported pain [55,56].

### 3.2. Assessment of Orofacial Somatosensory Function

The assessment of somatosensory function within the distribution of pain plays a central role in the certainty of the TMD diagnosis [57,58,59]. In this context, the QST is a psychophysical test procedure used to quantify the functional state of the somatosensory system of a patient by means of calibrated, graded innocuous or noxious stimuli and subjective perception thresholds [60]. Furthermore, QST comprises a battery of somatosensory tests assessing the response to a variety of standardized noxious and innocuous stimuli in affected and neighboring regions [61]. The feasibility of adapting the protocol to the orofacial region has been demonstrated recently [62]. Specifically, it has been shown that all 13 somatosensory tests can be performed on the apex of the tongue and facial gingiva in the upper jaw with moderate to excellent reliability for most measures. The duration of the intraoral examination per test site is in the range of 35 min, which is a bit slower than on extraoral sites [57].

### 3.3. Temporomandibular Disorders, Primary Headaches, and Cervical Pain

Painful orofacial and neck comorbidities are often associated with the TMD [63]. These coexisting conditions (particularly, headache, migraine, and neck pain) are not only highly associated with chronic pain-related TMDs but also increase the risk of their development [38,64,65]. The International Classification of Headache Disorders (ICHD) [66] and the DC/TMD [1] consider the main characteristics of pain in headaches and TMD, respectively. There are several hypotheses attempting to explain the association between TMD and headaches, including neuronal convergence, central sensitization, and inhibition of the descending pain downregulation mechanisms [67,68]. The strict relationship between TMD, headaches, and neck pain has been recently evaluated, not only in terms of sharing common pathogenic mechanisms and clinical features but also considering that one condition might influence or promote the development of another [11,64,69]. These conditions can cause facial pain and are frequently associated with the development of craniofacial allodynia during painful exacerbation [12]. Indeed, pain in both conditions has been attributed to common dysfunctions of the central pain regulation mechanisms [70,71]. On the other hand, the concomitance of TMD and migraines has shown worse levels of hyperalgesia and cutaneous allodynia, probably due to the sensitization of the central and peripheral nervous systems and the impairment of the descending modulatory pain pathways [44,70].

### 3.4. Evaluation Tools for Central Sensitization

The central sensitization should be adequately assessed, and one of the main evaluation scales in this field is the Central Sensitization Inventory (CSI) [72]. It includes two parts: part A, comprising 25 symptoms, such as physical symptoms, emotional distress, headache/jaw symptoms, and urological symptoms, and part B, consisting of 10 diseases, to evaluate central sensitization syndrome (CSS) [72]. A 40-point cut-off score of CSI part A was recommended to classify the presence of CSS in patients with chronic pain [73]. CSI development was based on a group of chronic pain conditions that has, probably among other mechanisms, central sensitization as a common putative pathophysiological mechanism [22]. The CSI has excellent test–retest reliability and internal consistency; the clinical and experimental characteristics of CSI could be commonly observed across many different chronic pain conditions, such as pelvic pain, osteoarthritis, spinal pain, and hereditary neuropathy [74,75,76,77]. Another evaluation tool is the Pain Sensitivity Questionnaire (PSQ), developed to assess various aspects of the clinical pain perceived [78]. The PSQ is a 17-item questionnaire that assesses a patient’s perceptions to various imagined physical stimuli that may be experienced in daily life [79]. Participants are asked to rate the pain intensity of each situational item on a 11-point scale, with 0 meaning “not painful at all” and 10 meaning “worst pain imaginable”. Three items (items 5, 9, and 13) are not normally rated as painful and are not included in the scoring. The PSQ-total score is the average of all items, except for the three nonpainful items. The PSQ-minor score is the average of items 3, 6, 7, 10, 11, 12, and 14—items, on average, that are perceived as causing minor pain. In patients with chronic localized pain, the PSQ-total and PSQ-minor scores have shown stronger correlations to QST compared to those found in healthy individuals [80].

Lastly, the Sensory Hypersensitivity Scale (SHS) is a 25-item instrumental tool assessing general and specific sensitivity and appears to be suitable as a screening instrument for central sensitization [81,82], albeit further studies should be performed to determine it in patients with TMD.

## 4. Evidence for Central Sensitization in TMD

To date, the scientific evidence on central sensitization in TMD in terms of diagnosis and treatment is still lacking.

In 2018, La Touche et al. [83] carried out a systematic review with a meta-analysis on central sensitization in TMD patients to summarize the scientific knowledge on this topic. The 22 included studies assessed the mechanical hyperalgesia (pressure pain thresholds), the thermal hyperalgesia (hot and cold pain thresholds), and the central hyperexcitability. Twelve studies evaluated the PPT, and eight of them revealed that PPT was significantly lower in TMD patients when compared to the control. Moreover, the meta-analysis showed strong evidence in favor of greater trigeminal pain sensitivity pressure in these patients. However, regarding thermal hyperalgesia, the meta-analysis comparing local and remote pain thresholds in patients with TMD and asymptomatic controls indicated that there were no significant differences in both cold and heat pain sensitivity between the groups. Lastly, spinal and central hyperexcitability were reported in TMD patients, as exhibited by increased mechanical temporal summation. Thus, the authors concluded that the findings of their systematic review and meta-analysis suggested the presence of peripheral and central nervous system sensitization in TMD patients.

However, the authors did not distinguish among different TMD subtypes, which are known to share different etiologies and pain mechanisms. Therefore, in 2021, Meng et al. [84] performed a meta-analysis to evaluate evidence in patients affected by only muscle pain-related TMD according to the DC/TMD [1]. The results provided evidence that, compared with the controls, these patients had reduced PPT and mechanical pain thresholds, whereas no evidence of changes in the cold detection threshold, warm detection threshold, heat pain threshold, cold pain threshold, and mechanical detection threshold were found.

Fernandez-de-las-Penas et al. [85] investigated bilateral, widespread pressure–pain hypersensitivity in nerve, muscle, and joint tissues in myofascial TMD women and controls. Their results showed significant differences between groups, but not between sides, for PPT levels over the supraorbital, infraorbital, mental, median, radial, and ulnar nerves; over the lateral pole of the TMJ; and over the tibialis anterior muscle. Thus, the results suggested both trigeminal and extratrigeminal sensitization of afferent inputs from neural tissues in myofascial TMD. An explanation could be related to the antidromic discharges originating from the central nervous system that may cause sensitization of peripheral nerve trunks that may depolarize nociceptive second-order neurons [85,86].

Additionally, in the scientific literature, it was shown that myofascial TMD patients presented larger referred-pain areas after intramuscular injection of hypertonic saline into the masseter muscle [87] and greater temporal summation of pain [88].

Furthermore, it should be noted that therapeutic strategies applied for managing central sensitization in TMD pain might be grouped into: bottom-up (i.e., tissue-based impairment treatments) and top-down interventions (i.e., strategies targeting the central nervous system) [89]. In this scenario, the bottom-up strategies could consist of joint-, soft tissue-, and nerve-targeting interventions, whereas top-down strategies might include physical therapy, motor imagery, and pain neuroscience education [89]. Therefore, we could conclude that, to date, the scientific literature showed that multimodal approaches seem to be more effective in patients with TMD, albeit it should be considered that the presence of depression might definitely increase pain sensitivity [90].

In this context, COVID-19 distress might have a negative impact on their psychological status, features of central sensitization, and facial pain severity in TMD patients [91].

## 5. Pharmacological Therapy and Central Sensitization in TMD

The purpose of pharmacological treatment of TMD is to alleviate the craniofacial pain linked to this condition, which is usually the main reason why these patients request medical attention [92,93]. Several therapeutic trials for TMD considered pain as the main outcome, evaluated with scales such as the visual analogic scale (VAS).

The mechanisms regarding chronic craniofacial pain remain to be fully elucidated [94]. Preclinical studies suggest that changes in both peripheral inputs and central brain structures could initiate and sustain chronic pain in TMD [94,95,96,97]. Inflammation with the release of many chemical mediators in TMD could result in the activation or peripheral sensitization of nociceptive endings of the trigeminal nerve [94]. Several mediators are linked to the peripheral sensitization, such as gamma amino butyric acid (GABA), serotonin, glutamate, and neuropeptides [94,95]. Conversely, the central sensitization strictly depends on an unbalance in the inhibitory and facilitatory descending pain modulatory systems, which promote and contribute to sustained chronic TMD pain [96]. Increased knowledge of those mechanisms underlying peripheral and central sensitization could improve pain management for TMD patients.

Pharmacological treatment for TMD pain could be very challenging for several reasons. First, as indicated above, multiple central and peripheral not yet fully elucidated mechanisms are involved in craniofacial TMD pain. Second, TMD pain is often associated with special emotional and psychological meanings, so a multidisciplinary approach could be indicated. Third, randomized controlled trials for the pharmacotherapy of these specific conditions are still lacking [98], so treatment is usually empirical. A Cochrane review evaluating TMD medications only included 11 studies in the qualitative synthesis [98]. The authors found lacking evidence to support or refute the efficacy of any drug for the treatment of TMD pain [98]. However, several medications typically prescribed in TMD have proven their effects for other craniofacial conditions.

The following subsections describe the most used drugs for the management of this condition, which are: nonsteroidal anti-inflammatory drugs (NSAIDs), beta-blockers, antidepressants, anti-seizure medications (ASMs), and opioids, along with other therapeutic approaches and new perspectives.

### 5.1. Nonsteroidal Anti-Inflammatory Drugs (NSAIDs)

NSAIDs are the most common class of medications prescribed for the treatment of craniofacial pain and have a proven efficacy for relief pain in TMD [99]. This class of medications include several molecules that exert their action inhibiting cyclo-oxygenase, thereby preventing the formation of prostaglandins. These drugs are usually well-tolerated and should be administrated for a minimum of two weeks to achieve an anti-inflammatory effect in TMD [100]. The main disadvantages of NSAID treatment could be: gastrointestinal adverse events, exacerbation of hypertension, and interactions with multiple drugs. The efficacy of topical or oral NSAIDs for the management of TMD pain is supported by controlled studies [101]. Ta and Dionne performed a six-week randomized double-blinded controlled trial comparing the efficacy of celecoxib, naproxen, and placebo for the treatment of TMD pain [101]. The authors showed that naproxen (500 mg twice a day) significantly reduced the clinical symptoms of TMD compared with celecoxib or a placebo [101]. De Carli and colleagues, in a double-blinded, randomized trial, showed that piroxicam, a cyclo-oxygenase-2 inhibitor, exhibited the lowest pain at a 30-day follow-up compared with the placebo [102]. Furthermore, Businco et al. showed that both topical and oral diclofenac are equally effective in the treatment of temporomandibular joint dysfunction symptoms [103]. One of the main advantages of the topical administration of diclofenac is preventing systemic adverse events of NSAIDs [103]. Topical creams of NSAIDs such as diclofenac could also reduce pain through the peripheral NMDA receptor antagonism [104]. Recently, a systematic review, including 11 randomized trails evaluating NSAIDs for the management of TMD, supported the use of NSAIDs in patients with TMD for the relief of pain [105].

### 5.2. Beta-Blockers

Beta-blockers exert their action by beta-adrenergic receptor antagonists. These drugs are widely used in other craniofacial conditions, such as for migraine prophylaxis [106]. Propranolol, a nonselective beta-blocker, is one of the most effective first-line drugs used for migraine prophylaxis [106]. The rationale for using beta-blockers to manage TMD pain comes from preclinical and animal models [107,108]. It was demonstrated that the activation of β₁ and β₂ adrenoceptors located in the TMJ region promotes serotonin (5-HT)-induced nociception [107,108]. To date, only few randomized controlled studies [98,99,100,101,102,103,104,105,106,107,108,109] have evaluated beta-blockers for TMD pain relief. A multicenter placebo-controlled trial using a “facial pain index” (FPI) as the primary endpoint evaluated the efficacy of propranolol in 200 TMD patients [109]. The authors showed that propranolol 60 mg twice a day was efficacious in achieving ≥30% and ≥50% FPI reductions after 9 weeks of treatment [109]. Tchivileva and colleagues showed a greater effect of propranolol in reducing FPI in migraineur TMD patients, suggesting that beta-blockers could be a reasonable option for treating the comorbidities of TMD and migraine [109].

### 5.3. Antidepressants

Both tricyclic antidepressants (TCAs) and selective serotonin reuptake inhibitors (SSRIs) have been reported to reduce pain in TMD patients. These molecules act by binding 5HT receptors, thus producing a significant modulation of the nociceptive system. TCAs, particularly amitriptyline and nortriptyline, have been extensively used for both the prevention of primary headaches and for the treatment of myofascial masticatory chronic pain [63,110,111]. Among various antidepressants, TCAs and SSRIs seem to be the most effective for chronic orofacial pain [112]. A placebo-controlled study with a 14-day follow-up demonstrated a significant reduction in pain and discomfort of TMD patients treated with 25 mg/day of amitriptyline compared with the placebo [113]. A systematic review suggests a type B level of recommendation in favor of using TCAs for TMD [114]. Among SSRIs, paroxetine, duloxetine, and citalopram have been used for treating TMD symptoms [115]. A recent study suggested a better outcome for TMD management when including a combination of duloxetine 30 mg twice daily and TMJ arthrocentesis [115]. Thus, we might conclude that doses of antidepressant drugs, if used to treat TMD pain, should be lower than those used to control depression symptoms.

### 5.4. Antiseizure Medications

Antiseizure medications (ASMs) were extensively used to treat neuropathic pain and primary headaches such as migraines. These drugs act at several sites of action, reducing neural hyperexcitability. The relevant sites of action include both voltage- and ligand-gated ion channels. Among several ASMs, gabapentin and pregabalin were extensively used for managing chronic facial pain [29,116]. These compounds were structurally related to GABA, the main inhibitory neurotransmitter in the central nervous system; in this context, ASMs could be considered as an alternative therapy for refractory TMDs [29]. Kimos et al. [116] randomized 44 patients with TMD pain either to take gabapentin or a placebo, demonstrating that gabapentin had a statistically significant effect over the placebo in reducing spontaneous pain in the TMJ and the number of tender sites on the muscles of mastication. Some ASMs such as clonazepam or diazepam belong to the class of benzodiazepines, which enhance the response to GABA by facilitating the opening of chloride channels and, thus, cause hyperpolarization. BDZ have an antiseizure, anxiolytic, muscle relaxant, and hypnotic effect; thus, they can modulate TMD pain at several levels. Harkins et al. conducted a 60-day double blinded, randomized trial, comparing clonazepam versus a placebo in the management of chronic TMD pain [117]. As argued by a Cochrane meta-analysis [98], clonazepam did not show a statistically significant difference compared to a placebo on pain in the right or left temporomandibular joints [117]. Another double-blinded clinical trial, in which patients with TMD pain were randomized to take clonazepam, cyclobenzaprine, or a placebo, failed to demonstrate statistically significant differences between clonazepam and cyclobenzaprine compared to the placebo on jaw pain [118].

### 5.5. Opioids

Opioids are a class of analgesic drugs, which act at the central and peripheral opioid receptors, resulting in the blockage of painful neural inputs. It has also been postulated on the existence of a peripheral m-opioid receptor event at the level of the TM joint, thus providing a rationale for their topical use in the management of TMD pain [119,120]. The most common prescribed opioids for oral administration are codeine and oxycodone, but their use is not recommended. If prescribed, they should be used for a short period only in patients complaining about severe TMD pain, refractory to other treatments [121].

### 5.6. Other Therapies and New Perspectives

Many other types of drugs are used for the management of TMD pain, such as corticosteroids or muscle relaxants such as cyclobenzaprine [122]. A meta-analysis showed that cyclobenzaprine could ameliorate TMD muscle pain in the short term through its effect on local spasms and the associated acute pain [123].

As demonstrated above, multiple oral drugs are quite effective in the management of TMD pain; however, systemic adverse events for oral drugs raise issues for any long-term treatment strategy. Future perspectives include novel delivery systems for therapeutic and regenerative agents to obtain satisfied clinical outcomes [124]. Minimally invasive delivered approaches containing biomaterials, cells, and/or bioactive molecules could complement classic pharmacotherapy, thus finally resulting in pain relief and improving joint function.

Lastly, both a hyaluronic acid (HA) injection and a platelet-rich plasma (PRP) injection may have a remarkable efficacy in the treatment of TMDs. More in detail, as depicted by Harba et al. [30], HA and PRP injections provide greater improvement in patients with TMDs as compared to a HA injection alone.

## 6. Physical Therapy and Rehabilitation for the Central Sensitization in TMD

### 6.1. Physical Therapy

Several clinical protocols for interventions and control groups differ; randomized clinical trials (RCTs) of jaw mobilization or stretching conditioning for TMD muscle pain indicate improvements in pain and jaw mobility compared with education and transcranial direct current stimulation, as well as betterment in pain compared with stabilization splints [125,126,127,128,129,130]. Reviews of postural exercises present progress in TMD muscle pain and jaw mobility compared with education alone [28,126,131]. Moreover, combinations of jaw-strengthening and coordination approach, with mobilization and postural programs, enhanced jaw mobility, and reducing joint pain [132]. In this scenario, resistance training with isotonic jaw-opening exercises plays a key role in muscle pain relief and mandibular range of motion improvement [132]. The underlying mechanism appears to be an inhibitory effect on the Golgi tendon. Golgi tendons, located in the target muscle, are stretched by isometric contraction, inducing an inhibitory effect on the muscle activity through Ib muscle fiber [133]. Moreover, postural exercise is typically employed for cervical spine pain management, but it can also be applied in the orofacial region, relieving muscle symptoms such as pain, tension, and stiffness by the influence of the head and mandibular position [134]. It is believed that the incorrect head position can induce muscle pain due to the acceleration of muscle activity in the neck and jaw muscles, as well as postural reflexes [135].

### 6.2. Occlusal Splints

The difficult relationship between occlusal interferences and temporomandibular disturbance seems to be explained on an animal model, such as the NMDA antagonist MK801 can attenuate occlusal interference-induced hyperalgesia, which suggests that central sensitization mechanisms are involved in the maintenance of the occlusal interference–TMD association [136]. In fact, Xie et al. [136] reported that occlusal interference could directly cause long-term masticatory muscle response in a laboratory animal model. Whether this mechanism may account for cases of TMD in humans needs further investigation. In this scenario, there were no inflammatory cells present, but Substance P expression in masseter muscles of both sides peaked at day 5 and then gradually decreased to the level of the control [137,138]. Their study suggests that, although no evidence of muscle damage and inflammation was found, peripheral sensitization appears to be involved in the mechanism of the EOI-induced masticatory muscle response [139]. However, the peripheral sensitization of nociceptive neurons cannot fully account for the long-standing nociceptive responses of masticatory muscles; a central sensitization mechanism may also be involved [136,140].

### 6.3. Extracorporeal Shockwave Therapy

The radial ESWT is a pneumatic pressure physical agent modality with direct mechanical stimulation that develops the maximum energy on the skin surface and radially diffuses into the tissues that might be used for musculoskeletal pain relief [34]. Radial ESWT has been widely recognized as a biological modulator that results in the differentiation of mesenchymal stem cells, neovascularization, and release of angiogenetic factors [141]. To date, it is unclear how ESWT can affect temporomandibular disorders. Taking as valid the hypothesis of the mechanotransducive effect of ESWT in other diseases, it could be hypothesized that the waves at the level of the microcirculation can increase the perfusion, promote angiogenesis, and alter the signaling of pain in ischemic tissues caused by the influx of calcium [34,142]. On the other hand, recent articles have shown that free nerve endings degenerate after the application of ESWT and that ESWT produces a transient dysfunction of nerve excitability at the neuromuscular junction, resulting in the downstream of AChR [143]. Although this test was performed on spastic muscles, it could also be extrapolated to the MTP and the energy crisis hypothesis [144]. Lastly, following a purely mechanistic approach, shockwaves may be able to break actin-myosin bonds, as they propagate perpendicular to the sarcomere contractions [143].

### 6.4. Laser Therapy

Low-level laser therapy (LLLT) has recently been put under the spotlight, because the proponents claim its easy application, limited treatment time, and minimum contraindication. Theoretically, LLLT is a nonthermal type of light, thought to reduce inflammation through the increase of ATP production, improvement of local microcirculation, reduction of edema through an increase of lymphatic flow, and decrease of the prostaglandin E2 and cyclooxygenase-2 levels, albeit the mechanism underlying the therapeutic effects of LLLT is still under debate [145]. Actually, it has a complex mechanism of action, resulting in three main effects on tissues- through direct irradiation without causing a thermal response [146]. Biostimulation occurs through metabolic activation and increased vascularization and fibroblast formation, while the anti-inflammatory and analgesic effects of LLLT are probably due to multiple actions [147,148]. It increases the beta-endorphin level in spinal liquor and increases the urinary excretion of glucocorticoids, which are inhibitors of the synthesis of beta-endorphins [149,150]. It also increases the pressure pain threshold through a complex electrolytic nerve fiber-blocking mechanism and causes a decrease in the release of histamine and acetylcholine and a decrease in the synthesis of bradykinin [151].

### 6.5. Transcutaneous Electrical Nerve Stimulation

TENS is defined as the application of electrical stimulation to the skin for pain control. It is a well-known form of physical therapy, which is useful for the relief of pain. It is a safe, noninvasive, effective, and swift method of analgesia, and the potential adverse reactions of other methods of pain control are eliminated [152]. Particularly, at the spinal level, low-frequency low-amplitude TENS works on *µ* receptors, while high-frequency high-amplitude TENS works on *δ* receptors. Spinal administration in an animal model of a low dose of naloxone (at a low dose, naloxone works as a specific antagonist of the *µ* receptor of endogenous opioids) and naltrindole (antagonist of *δ* receptors) in arthritic rats prevented anti-hyperalgesia after both low-frequency low-amplitude and high-frequency high-amplitude TENS showed that the *δ* and *µ* receptors were the target of the stimulation [153,154]. Moreover, a particular type of TENS has been used for a long time in dentistry for a variety of purposes, ultralow-frequency TENS (ULFTENS), because of the frequency of the stimulation (0.66 Hz) belonging to the field of ultralow frequencies (<20 Hz) [155]. In the “normal” condition, collaboration exists for the control of arousal between the cortical and subcortical centers [156]. Information transmitted through sensory ULFTENS reaches the nuclear trigeminal sensory complex and, through the latter, is projected to the subcortical areas that control arousal. Acute stress and pain lead to increased arousal (allostasis), followed by the temporary activation of peripheral responses mediated by the autonomous nervous system, as well as the inflammatory, immune, hormonal, and neuromuscular systems [157,158]. It is probable that such action takes place by the “inhibition of the inhibition” of the “activation system”, according to the hypothesis of Thayer [24]. Thus, ULFTENS acts through the balance of the subcortical arousal circuit by enhancing the inhibition through the endorphin system and reducing the cortical activation induced by stress or pain [24].

### 6.6. Biofeedback

In recent years, the usefulness of biofeedback therapy in patients with different muscle disorders, including TMD, was suggested. Florjanski et al. [28] conducted a systematic review to evaluate the efficacy of biofeedback and concluded that the majority of the included studies presented a significant correlation between biofeedback usage and the reduction of muscle activity.

## 7. Interventional Therapies and Central Sensitization in TMD

### 7.1. Acupuncture and Dry Needling

Since the introduction of acupuncture therapy into modern Western medicine, numerous studies have been carried out to investigate and explain the scientific basis behind it [159]. The arrival of qi or “de qi” refers to the transmission of a needling sensation along the meridians, which is often described by the patients as soreness, numbness, fullness, warm sensations, or aching as a result of needle manipulation [160]. Recent histological evidence using rat models seems to suggest this needle grasp sensation is the result of collagen and elastic fibers tightening around the needle during needle manipulation [161]. The authors went further to postulate this mechanical coupling between the needle and soft tissue as being responsible for transducing mechanical signals into fibroblasts and other cells, with resultant therapeutic downstream effects [160,162]. The authors proposed that the stimulation of acupuncture points can relieve pain by causing “hyperstimulation analgesia”, which can be explained by the concept of the “gate control theory of pain”, proposing that the activation of A-δ and C afferent fibers through acupuncture point stimulation sends signals to the spinal cord with a local release of dynorphin and enkephalins [162]. In this scenario, neurotransmitters such as serotonin, dopamine, and norepinephrine are produced, causing the pre- and postsynaptic inhibition of pain transmission, and when the signals reach the hypothalamus and pituitary gland, adrenocorticotropic hormones and endorphins may be produced [163].

### 7.2. Botulinum Toxin

Botulinum toxin (BoNT) is the protein group produced by anaerobic bacteria called *Clostridium botulinum*, which has approximately 40 subtypes. However, seven serotypes are typically noted based on antigen specificity. BoNT-A has been the subject of innumerous studies to confirm its antinociceptive effect [164]. Indeed, for a long time, the analgesic effect of BoNT type A (BoNT-A) was considered to be due to the effect of muscle relaxation, particularly in the case of stroke spasticity [165,166]. However, BoNT has been used for neuropathic pain with an analgesic effect independent of muscle relaxation by demonstrating dissociation of the duration of muscle relaxation and duration of pain relief [167,168]. More in detail, the reduction of inflammatory hyper-nociception may be due to an inhibition in the release of certain pain-related neurotransmitters and proinflammatory cytokines [169]. Moreover, the BoNT-A mechanism of action could be not only restricted to a peripheral mechanism but also to a central action on three neurotransmitters: SP, CGRP, and glutamate (Glu), where the inhibition of Glu release takes on a more important role than the one earned peripherally [170]. In vitro models, using cultures of embryonic rat dorsal root ganglion, demonstrated BoNT-A inhibition of SP release and the reduction of stimulated CGRP [171]. Specifically, BoNT-A can directly decrease the amount of CGRP released from trigeminal sensory neurons in cultures of rat trigeminal ganglia. CGRP is a multifunctional regulatory neuropeptide strongly related in the underlying pathology of migraines [172]. In addition, it was found that albumin-induced arthritis increased the release of the proinflammatory cytokines IL1-β and TNF-α [173]. Despite these studies providing important contributions to the better understanding of the antinociceptive mechanism of BoNT-A, more experiments to elucidate this effect are necessary [168].

### 7.3. Oxygen–Ozone Therapy

Oxygen–ozone therapy is an adjuvant treatment that plays an anti-inflammatory and analgesic effect in several pathological musculoskeletal disorders characterized by chronic inflammatory processes (e.g., low back pain, osteoarthritis, cervical pain, fibromyalgia, and TMD) [36]. The effect of oxygen–ozone therapy mimics an acute oxidative stress that, if properly balanced, is not harmful but is able to provoke positive biological responses and reverse chronic oxidative stress (degenerative process, aging, etc.) [174]. This hypothesis about ozone and oxidative stress modulation could be better defined as a “real non-toxic therapeutic shock able to restore homeostasis” [175]. Low doses of oxygen–ozone could therefore play a role in the regulation of prostaglandin synthesis, in the release of bradykinin, and in the increase of macrophage and leukocyte secretions. It is widely accepted that pain is a common symptom related to the inflammatory process, and oxygen–ozone therapy could play a key role not only in the management of inflammation but also in nociceptive perception and modulation [176]. As for the analgesic use, after the administration of oxygen–ozone, an increase in the antioxidant molecules (serotonin and endogenous opioids) has been demonstrated, which would induce pain relief by stimulating the antinociceptive pathways [177,178].

## 8. Study Limitations and Strengths

In conclusion, the diagnosis was a fundamental starting point to comprehend the pathology of these subjects; indeed, although all of them presented craniofacial pain, it varied depending on the origin: muscular, arthrogenous, or a combination of them. Chronic craniofacial muscle pain associated with TMD involves multiple peripheral and central mechanisms, and it should be taken into consideration that the coronavirus pandemic has caused significant adverse effects on their psycho-emotional status, resulting in the intensification of their bruxism and TMD symptoms and thus leading to increased orofacial pain [179,180].

This study is not free from limitations, such as the lack of systematic research of the literature and the absence of a meta-analysis. However, the study heterogeneity might not allow a quantitative analysis, in accordance with the Cochrane Handbook for Systematic Review of Intervention (Ver, 6.2, 2021) [181]. Furthermore, it could be difficult to draw strong conclusions starting from so wide a presence of observational studies with different outcomes measured, testifying to the difficulty in the assessment of the central sensitization.

On the other hand, it should be noted that this comprehensive review is the first in the scientific literature investigating both the diagnosis and treatment of patients with TMD by adequate control of the central sensitization. The resulting evidence showed that several pharmacological and conservative approaches could have a potential effective role in the regulation of the central sensitization in patients affected by TMD pain.

## 9. Conclusions

Taken together, the findings of the present comprehensive review showed that the central sensitization and the inhibitory system of descending pain might play a role in the TMD clinical pattern. In this context, pharmacological drugs and conservative approaches (e.g., occlusal splints, ESWT, LLLT, TENS, and oxygen–ozone therapy) could have a positive impact in terms of the central sensitization of TMD pain. Further observational studies should investigate the role of these rehabilitative approaches in pain relief in patients affected by TMD.

## Data Availability

Not applicable.
